# Biography of Claude Bernard

**Published:** 1860-06

**Authors:** 


### SELECTIONS.

(From the London Medical Times and Gazette.)

*Biography of Claude Bernard.*—The intensely interesting and highly instructive Lectures on Experimental Pathology and Operative Physiology, which have been recently commenced at the College of France, by M. Claude Bernard, being about to appear in the columns of the *Medical Times and Gazette*, a brief notice of the labors and scientific career of their distinguished author may not be without interest for the reader.

M. Claude Bernard was born in 1813, at St. Julien, near Villefranche, in the department of the Rhone. I am unable to state exactly in what year he commenced his medical studies, but it must have been about '34 or '35, for, in 1839, he, after undergoing the customary ordeal, entered one of the Paris Hospitals as "*int-rne.*"

Two years later he became attached to the lecture-room of the celebrated Magendie, at the College of France, his position being that of "preparateur." In other words, the duty devolved on him of making all the preliminary arrangements which the proposed experiments of that distinguished professor might require.

In 1843, the youthful Bernard, after a brilliant examination, and the usual defence of a thesis, was received as M. D.; and in 1853, he obtained the degree of "Docteur en Sciences,"—no mean honor, as all those who know the severity of the test must admit.

In 1847 we find him occupying the honorable and important office of "suppleant," or substitute to Magendie, and even at times lecturing with very considerable ability to the crowds of scientific men and students who were wont to repair to the lecture-room of that distinguished man. This office, of such high trust and responsibility, he worthily held for seven years.

The natural bias of his mind had, from the very commencement of his studies, inclined him towards physiological researches; but, alas! Bernard was not one of fortune's favorites, and his scanty means forced him to quit the field where he was destined, at a later period, to gain such glorious laurels, and to return to the domain of Surgery. He even went so far as to publish a "*Manuel de Medecine Operatoire*," in collaboration with M. Huette. Circumstances, however, having brought him in contact with Magendie, the marked taste which he speedily evinced for physiology satisfied that great man that he might one day be surpassed by the young aspirant. Fortunately for science, Magendie possessed great influence over him, and succeeded in calling him back to his less lucrative but more favorite studies of physiology.

Some short time after this backsliding—if I may be allowed to use the expression—he was called upon to occupy a position of higher importance still, and one more consonant with his independent and speculative nature than that of assistant to another could possibly be. I allude to the chair of physiology, which has just been created, in connection with the Faculty of Sciences.

But higher honors were in store, and thick and fast did they descend on him; for we find that, shortly after having attained to the Professorship, he was elected a Member of the Academy of Sciences, in lieu of M. Roux, the eminent surgeon, whose death has just caused a vacancy in that learned body.

The following year was signalized by an event which profoundly moved the scientific world; namely, the death of Magendie, whose name had been for years identified with the progress of experimental physiology, and who had by his extraordinary success earned for himself the name of "Chief of the Experimental School of Physiology of France." It was well known that the end and object of Magendie in all his teaching and investigations was the subjugation of theory to practice; and in this respect he was a most valuable guide and director to those who were disposed to follow him in his experiments. Skeptical and inquisitive by nature, he mercilessly overthrew whatever would not stand the test of experiment. From such a master the inquiring mind of Bernard could not but take a favorable bias; from such a man he could not fail to draw healthy inspirations. Hence we find Bernard adopting the principles of his esteemed master, and steadily and perseveringly improving and enlarging the field of experimental science—philosophically considering and investigating the normal and morbid manifestations of the animal economy and the laws of life. It was but natural to suppose that the illustrious Magendie should be replaced by his talented pupil; and right worthily has he since filled up the blank which his master's death created, as the attentive and admiring crowds always to be seen in his class-room amply testify. It is not the orator they flock to hear; for as a speaker we daily hear better. So rapidly do his ideas seem to succeed each other that he is often at a loss to find words to clothe them. His voice, though not harmonious, is far from being unpleasant. In stature he is above the middle size, well knit, broad-chested, of a nervo-bilious temperament—the latter element predominating. A highly intellectual expression of countenance, with a large and powerful head, give unmistakable evidence of the energy and indomitable perseverance of the man. Though not a rhetorician, in the strict sense of the word, he possesses the rare and happy talent of captivating and enchaining his audience, and inspiring them with the conviction that he is fully and completely master of the subject which he expounds.

But to take a glance at his labors, and what he has already achieved in his particular department. Almost all of his discoveries are of a highly important and practical kind; and they have given, within the last few years, quite a new character to physiological investigation. He has not only struck out new paths, but he has roused the attention of the scientific and the learned to the reconsideration of many fundamental questions which were supposed to have been long settled, but which, in reality, had been but imperfectly established; and he has thereby contributed much to a clearer, a more correct, and a more comprehensive appreciation of the essential functions of the animal economy. As far back as 1844, when he was comparatively a young man, and but newly entered on the field of physiological investigation, he published an elaborate paper on the different secretions of the alimentary canal, and the parts which they respectively play in the digestive process. He had the merit of being the first to show the real mechanism of the secretion of the gastric juice, and the various changes and modifications produced by this liquid on the aliments taken into the stomach. Not less interesting and instructive are the results of his investigations into the saliva and the intestinal secretions generally, and his inquiries into the influence of the different pairs of nerves on the organs of digestion, circulation, and respiration.

But it was in the year 1849 that Bernard first laid the real foundation of his reputation as an experimental physiologist. Prior to this period the real function of the pancreas was involved in obscurity. It had been considered in the light of a salivary gland—a conclusion derived from the similarity of its structure to organs of this class. By a series of carefully conducted experiments, Bernard showed most conclusively that the real function of the pancreas related to the formation of chyle and the digestion of fatty matter taken into the stomach. For this important discovery he was honored with the great prize for Experimental Physiology, awarded by the Academy of Sciences in that year.

In 1850, he made known to the scientific world his first discoveries in connection with the liver; and he showed that this organ—the principal use of which in the animal economy was believed to be the secretion of bile—had, in reality, another important function, the existence of which had been, up to this time, completely ignored by physiologists. This discovery was no other than that the liver, in its normal condition, besides secreting bile, was constantly producing sugar. To this new function he gave the name of *Fonction glycogenique du foie.* By an immense number of experiments, conducted on species belonging to three of the principal branches of the animal king dom, he proved to the entire satisfaction of the Academy of Sciences that the blood, before entering the liver by the venæ portæ, contains no sugar; while that which leaves the liver, to enter the heart by the hepatic veins, is abundantly charged with this element. He further proved that this new function was intimately connected with and influenced by the nervous system, and that, by operating on the latter at certain points, an artificial diabetes mellitus can be produced at will. This important discovery, which at first met with much opposition, is now, so far as I know, an acknowledged fact; and its importance, as regards the pathology and treatment of diabetes, is too evident to require remark. It follows from it, that this malady is nothing more nor less than the disturbance of a physiological function; and, that function residing in the liver, it is to this organ, and to those parts of the nervous system which influence it, that the medical man must direct his attention, with a view to its cure. For this most important and practically useful discovery, M. Bernard was again awarded the great prize for Experimental Physiology.

In 1851, his researches in connection with the great sympathetic were so highly approved by the Academy of Sciences, that, for the third time, he received the great prize in physiology. They have since been published, and are not the least interesting of his numerous productions. He shows therein, that if a section be made of any of the branches of this nerve, the temperature of the parts which they supplied is instantly and permanently augmented, and that the inverse of this takes when the nerves of the cerebo-spinal axis are devided—in other words, that, in this latter case, there is a manifest diminution of the temperature. Further, that the section of branches of the great sympathetic, besides being followed by increased temperature, is also attended with great vascularity of the parts which these branches supply. It is easy to appreciate, in practical medicine, the great value of these discoveries, which, up to the present time, so far as I am aware, have not been controverted.

Other discoveries on the subject of animal heat, too numerous to be embraced in this notice, have also been made known by M. Bernard. His experiments proving the elective elimination of certain substances by the secretions, and especially by those of the salivary glands, as well as his discoveries on the special functions of the spinal nerves, are fraught with intense interest and importance, as well to the physiologist as to the practical physician. Indeed, there is hardly a question in the wide domain of physiology and pathology which has escaped his attention.

Having thus touched on the leading points in M. Bernard's scientific life, we must not forget to add that he follows science for science's sake; patiently and perseveringly he toils for seven or eight hours every day in his laboratory. The world deeply indebted to him; and, nevertheless, he is but poorly remunerated. His two professorships—the one at the Faculty of Science, and the other at the College of France—together with the trifling sum derived from the Institute, of which he is a member, constitute in all but a modest income—not greater, perhaps, than that of a moderately busy country practitioner in England. Thus is science honored! thus are its disciples recompensed in military and imperial France!

Before concluding this paper, it may be well to say a few words on the College of France. This institution was founded by Francis I. in 1530, at the joint solicitation of the preacher Parvi and the famous Beaudens. The number of professors, which was at first but limited, amounts now to twenty-eight. These professors—or "Lecteurs," as they were originally named, from their duty having been, in early times, to read classical authors to the students—give lectures on all the leading subjects in science, literature and art. One peculiarity in this college consists of the perfect liberty accorded to the teachers in their several departments. For example, the Professor who occupies the chair of medicine, has the privilege of teaching any one of the numerous branches of medical science. He may lecture on surgery, materia medica, therapeutics, physiology, or any other subject embraced under the general head medicine.

The edifice is plain, but elegant. Among other apartments, it contains some eight ampitheatres, where lectures are delivered. In several of these certain professors lecture by turn. That used by M. Bernard is exclusively set apart for the chair of medicine. It is a large square room, capable of containing six hundred students. At one side of the room, on an elevated platform, is the professor's chair, immediately in front of which is a table, some ten or twelve feet long, on which all the experiments conducted in public take place. From the front of this platform the seats for the students rise in tiers. The roof is ornamented with four frescoes, representing Hippocrates, Aristotle, Buffon and Limnæus. Elegant as is the general appearance of the room, it has a serious defect: the light being derived from the roof, falls directly on the table, and any delicate operation, requiring close inspection, forces the professor to place his head in a position which effectually intercepts the rays of light on their way to the object under examination. In an adjoining apartment is the laboratory, which consists of two small rooms. In that nearest the lecture-room are some small furnaces, and sundry glass cases, containing the larger instruments required for the experiments. In the centre of this room is a strong, solid table about five feet by three, perforated in sundry places, so as to permit cords to pass through it, to control the movements of the animals subjected to vivisection. The other room resembles a chemist's shop. In it are kept all the chemical and medicinal agents, as well as the smaller instruments. In one corner is a sand-bath, intended for experiments on cold-blooded animals. Beneath these apartments, and connected with them by a stone staircase, are a series of cellars, dark and dismal enough, in which are kept animals of every description—dogs, rabbits, guinea-pigs, etc., etc.,—with here and their huge basins and troughs, filed with frogs and other cold blooded animals—all intended in their turn to be sacrificed and offered up on the alter of science. Although that part of the College of France in which M. Bernard lectures is modern, as compared with the rest of the building, still it leaves much to be desired. The laboratory is too small; and it is a matter of wonder to those who visit it, how the professor, his immediate assistants, and his numerous private pupils, can move about in the pursuit of their studies. It is to be hoped that an amelioration, in this respect, may, erelong, be effected.

